# Analysis of Spatiotemporal Features in a Virtual Navigation Game Across Different Age Groups: Quantitative Research

**DOI:** 10.2196/83128

**Published:** 2026-04-02

**Authors:** Xiaofeng Qiao, Shan Tian, Min Tang, Shipei He, Jinghui Wang, Linyuan Fan, Yuanjie Zhu, Zhiyang Zhang, Songjun Du, Chaojie Dong, Yepu Chen, Xiaoyu Liu

**Affiliations:** 1 School of Biological Science and Medical Engineering, Beihang University, 5 Building in Beihang University, 37 Xueyuan Road, Beijing, China, 86 1082339861; 2 Key Laboratory of Biomechanics and Mechanobiology of Ministry of Education, Beihang University, Beijing, China; 3 Key Laboratory of Innovation and Transformation of Advanced Medical Devices, Ministry of Industry and Information Technology, Beijing, China; 4 National Medical Innovation Platform for Industry-Education Integration in Advanced Medical Devices (Interdiscipline of Medicine and Engineering), Beijing, China

**Keywords:** spatial navigation, environmental structure, serious games, space syntax, cognitive assessment, urban planning and design, aging

## Abstract

**Background:**

With rapid urbanization, the proliferation of densely arranged buildings and increasingly homogeneous architectural designs has made disorientation and navigation difficulties more common, especially for older adults. Meanwhile, advances in virtual reality technology now allow researchers to create highly immersive navigation games, offering opportunities for assessing cognitive abilities and examining how environmental factors shape navigation behavior.

**Objective:**

This study aimed to design a virtual reality–based navigation game capable of assessing cognitive abilities through navigation behavior and quantitatively examining how environmental configurations influence navigation patterns in different age groups.

**Methods:**

We designed a virtual goal–directed navigation game and recruited 2 groups, younger adults (n=18) and older adults (n=21), to complete identical wayfinding tasks. Before the formal experiment, participants completed cognitive assessments and received training. To characterize navigational behavior, k-means clustering was applied to classify navigation states and extract behaviorally meaningful navigation measurements, which were then examined for correlations with cognitive test scores. To quantify the effects of environmental structure, space syntax analysis was conducted to calculate line-based and grid-based experienced metrics for each participant, and their associations with navigation performance were examined. Additionally, between-group differences in navigation performance and experienced metrics were evaluated across age groups.

**Results:**

Our results revealed that navigation behavior performance, particularly navigation efficiency, was significantly influenced by cognitive abilities and was strongly associated with several cognitive tests: the Montreal Cognitive Assessment (*r*=0.495, *P*=.04), Trail Making Test Part A (*r*=−0.761, *P*=.001), and the Mental Rotation Test (*r*=0.848, *P*<.001). In terms of environmental influences, experienced axial integration (EAI) and experienced visual integration (EVI) demonstrated significant age-related differences: EAI (*z*=–2.43, *P*=.01) and EVI (*t*=2.48, *P*=.02). Moreover, navigation efficiency exhibited distinct age-specific correlations with experienced metrics: among older adults, navigation efficiency was negatively associated with EVI (*r*=–0.48, *P*=.04), and young adults showed negative correlation between navigation efficiency and EAI (*r*=–0.64, *P*=.005).

**Conclusions:**

Our findings demonstrate that k-means clustering provides an effective approach for classifying navigation states and extracting quantitative behavioral indicators for assessing cognitive abilities. In addition, the environment-based experienced metrics derived from space syntax analysis revealed distinct age-related navigation patterns, highlighting how spatial configuration shapes wayfinding behavior across age groups. These results establish an important foundation for future applications in clinical cognitive assessment and rehabilitation, as well as the design of age-friendly urban environments.

## Introduction

With rapid urbanization, the proliferation of densely packed buildings with homogeneous architectural designs has increased the likelihood of disorientation and navigation difficulties. Irregular street networks and the frequent modification of road layouts further disrupt the formation of cognitive maps and hinder spatial orientation [1]. Spatial navigation, the ability to determine an appropriate route and purposefully move toward a specific destination, has increasingly become a considerable challenge, particularly for older adults [2,3].

Significant declines in navigation ability have been recognized as one of the earliest indicators of mild cognitive impairment and Alzheimer disease [4,5]. Individuals experiencing navigation difficulties tend to confine themselves to familiar environments, resulting in critical limitations on their ability to engage in daily activities [5]. Neuroscientific evidence underscores the vulnerability of the entorhinal cortex, a crucial region involved in spatial navigation, to neurodegenerative processes [6]. Grid cells within the entorhinal cortex, which are essential for spatial representation and path integration, exhibit notable functional impairments as individuals age. These age-related deficits in grid cell activity may disrupt the formation of stable cognitive maps, leading to difficulties in orientation, route planning, and wayfinding [7]. Early and rapid identification of navigation impairments is of critical importance.

Evidence from previous studies has indicated that behavioral performance in navigation tasks is influenced by both extrinsic factors (eg, spatial properties of the environment, visual accessibility of the landscape) [8,9] and intrinsic factors (eg, age, cognitive abilities) [10,11]. These factors jointly determine how individuals gather and interpret spatial information, select navigation strategies, and execute wayfinding decisions. Extensive research has demonstrated that environmental cues [12], such as the configuration of landmarks [8], locations [13], and paths [14,15], play a critical role in guiding navigation behavior as well as the neural processes underlying spatial orientation [16,17]. Importantly, age-related differences in the use of environmental cues are well documented [18]. Older adults tend to rely more on associating specific directional responses with particular landmark stimuli [19]; when geometric cues conflict with landmark cues, they are more likely to maintain the landmark-response association [20]. This reliance reflects an egocentric navigation strategy, in which spatial relations are encoded from a body-centered perspective, and actions are guided by subject-to-object associations [18,21,22]. In contrast, younger adults more effectively integrate both geometric and landmark cues, showing greater flexibility in switching between allocentric (map-based) and egocentric (route-based) frames of reference [18].

Although many studies have explored how modifying environmental elements affects navigation behavior, a consistent and intuitive way to characterize spatial properties across different settings is lacking. Space syntax, a well-established framework for quantifying spatial configuration [23], offers a powerful solution. It has been widely applied in urban design [24], path planning [25], and wayfinding research [26]. Within this framework, axial map analysis (AMA) [13] characterizes spatial structure through line-based representations, whereas visibility graph analysis (VGA) [9,27,28] captures grid-based visibility relationships. Key parameters, including integration, depth, and connectivity, reflect how accessible, shallow, or interconnected different spatial segments are. Prior research has demonstrated the value of these metrics for predicting exploration and wayfinding behavior. Among them, integration is particularly important, as it indicates how accessible a location can be reached from all others and is considered a key determinant of navigation difficulty [29]. Moreover, experienced integration has been shown to indicate the extent to which individuals concentrate their movement within globally integrated regions [13]. Spending more time in highly integrated areas enables individuals to form more accurate cognitive maps [14], resulting in higher experienced integration. Building on this theoretical foundation, we further introduce a series of experienced metrics derived from both axial and visual analyses to quantify the environmental structures that influence participants’ actual navigation behavior.

Recent advancements in virtual reality (VR) technology have enabled the creation of highly immersive virtual environments (VEs) that closely replicate the real world with high fidelity [30,31] and have been widely applied in the field of spatial navigation. Researchers can precisely control environmental variables such as landmark distribution, path complexity, spatial layout, and visual cues in immersive VEs [32], making it possible to investigate navigation behavior under the influence of different factors. Moreover, VR technology allows for the collection of a wide range of behavioral data, such as completion time, movement trajectories, error rates, and specific behavioral reactions at given moments [33]. By constructing immersive VEs and a task framework, researchers have developed a variety of experimental paradigms designed for specific navigation tasks, including the Virtual Floor Maze Test [34], Morris Water Maze task [35], and object-location memory task [7,36]. Efficient VR-based navigation tasks are regarded as valuable tools for assessing and enhancing spatial navigation [34].

In recent years, navigation tasks have increasingly been gamified [37,38], with successful applications reported in both the health care domain and geographic science research. For example, the mobile game Sea Hero Quest has been widely used for large-scale assessment of human navigation ability [39], providing detailed analyses of navigation performance across age groups and cultural backgrounds [12,40-42]. In parallel, gamification approaches combined with location-based services have demonstrated effectiveness in improving users’ understanding of urban maps, orientation, and wayfinding skills in real-world city environments [43,44]. The introduction of serious games (SGs) has enhanced the engagement of navigation tasks while preserving scientific validity. By incorporating clearly defined goals, standardized task structures, and meaningful feedback within immersive environments, SG-based VR navigation paradigms provide effective platforms for assessment, training, and rehabilitation [45].

In this study, we intentionally integrated structured navigation tasks into a goal-oriented game framework. We developed a goal-oriented VR-based navigation game, aimed at effectively assessing individual cognitive abilities and exploring how environmental configurations influence navigation behavior. Specifically, the objectives were to (1) evaluate the feasibility and reliability of the VR navigation game as a cognitive assessment tool; (2) compare navigation behaviors between younger and older adults; and (3) examine how key environmental factors shape navigation performance across age groups. These findings are expected to provide methodological support for cognitive assessment and training, as well as empirical evidence to inform spatial planning in aging cities.

We hypothesized that navigation behavior would be closely associated with cognitive abilities, such that individuals with higher cognitive capacity would demonstrate more efficient wayfinding performance. We further expected that younger and older adults would exhibit distinct navigation patterns, reflecting age-related differences in cognitive processes and strategy use. Finally, we anticipated that quantitatively measured environmental configuration would exert effects on navigation efficiency, and that these effects would differ significantly between age groups.

## Methods

### Ethical Considerations

This research involved human participants. Approval of all ethical and experimental procedures and protocols was granted by the Ethics Committee of Beihang University (BM20230063). Strict privacy protection measures were applied, and all data were anonymized with no personally identifiable information included. Prior to participation, all individuals were fully informed about the study procedures, potential risks, and their right to withdraw at any time without penalty. All participants provided written informed consent and were compensated at a rate of 100 CNY (approximately US $14) per hour.

Considering that older adults constitute a potentially vulnerable population, additional safeguards were implemented for the older adult group. These included the presence of at least one qualified health professional to provide immediate medical support, repeated verbal reports to monitor fatigue, dizziness, and overall comfort, and the option for participants to terminate the experiment immediately upon experiencing any discomfort.

### Participants

The study was conducted with 21 healthy older adults (14 males and 7 females) aged 65 to 80 years (mean age 71.79 , SD 4.57 years) and 18 healthy younger adults (10 males and 8 females) aged 21 to 33 years (mean age 26.50, SD 2.99 years). All participants had no history of neurological or psychiatric disorders. Older participants were recruited from the community and were at least 65 years old and able to complete daily activities independently. One older participant with a history of cerebral hemorrhage was excluded prior to the study, and another withdrew due to intolerance to VR-induced dizziness. Younger participants were recruited through public advertisements posted on the university campus. Individuals who expressed interest were screened using the same inclusion criteria applied to all participants, including normal or corrected-to-normal vision, no history of neurological or psychiatric disorders, and no prior diagnosis of cognitive impairment. Additionally, individual information, encompassing age, gender, education, and prior VR experience, was systematically gathered from each participant.

### System Configuration

The virtual navigation game used in this study was developed with Unity 2019.4 (Unity Technologies) and deployed via the HTC VIVE Pro head-mounted display. The visual scene was rendered at 90 Hz and presented at a single-eye resolution of 1440×1600 pixels (2880×1600 binocular) with a 110° field of view, providing an immersive experience. For the experimental setup, the head-mounted display was connected to a high-performance desktop PC via a wired connection using the standard VIVE link box to ensure low latency and stable rendering. The system was equipped with an Intel Core i7-9700K CPU, an NVIDIA GeForce RTX 2070 GPU, 32 GB of RAM, and the Windows 10 operating system, enabling smooth rendering and reliable behavioral data acquisition.

The VE was designed as a simplified yet ecologically valid urban block (200 vm×150 vm, virtual meters), featuring residential buildings, street networks, greenery, and small parks. The intentional integration of these elements provided sufficient spatial complexity to elicit meaningful wayfinding behavior while minimizing potential discomfort or anxiety associated with navigation in a VE [46].

Given that older participants may experience limitations in sustained physical walking, we adopted a hybrid navigation control scheme combining the handheld controller and natural facing direction [47]. The VIVE controller features an ergonomic design with a circular touchpad. To minimize operational complexity, participants were instructed to press the touchpad to move forward in the direction they were facing, and releasing it stopped movement immediately. Horizontal body rotation was mapped one-to-one to virtual orientation, allowing participants to turn naturally without additional button input. Throughout the experiment, all participants were instructed to avoid movement in the physical space and to navigate using the controller and body rotation only. Most participants completed the game while standing, whereas three older participants, due to advanced age, performed the experiment while seated in a swivel chair that allowed 360° physical rotation. This simplified control scheme reduced cognitive and motor demands while ensuring consistent interaction across participants.

### Experimental Procedure

The experiment consisted of three phases: (1) neuropsychological assessment, (2) pretraining, and (3) formal experiment. The neuropsychological assessment phase aimed to evaluate the cognitive abilities of older participants using standardized measures of general cognitive skills and empirically validated tests. During the pretraining phase, participants were instructed to navigate freely within the VE for 15 minutes. Navigation proficiency was considered achieved when participants demonstrated the ability to independently and fluently operate the controller and navigate to designated target locations, qualifying them to proceed to the formal experimental phase. In the formal experimental phase, participants were required to sequentially navigate to multiple target locations, with spatiotemporal data recorded at fixed intervals throughout the navigation process. Older participants completed all 3 phases, while younger participants, who were healthy university students with presumed normal cognitive function, only participated in phases 2 and 3.

Before the formal navigation tasks, general cognitive functioning was quantified using the Mini-Mental State Examination (MMSE) [48,49] and the Montreal Cognitive Assessment (MoCA) [50]. Specifically, the MMSE administered in this study was the standardized Chinese adaptation based on the international MMSE framework, which has been validated through prior pilot testing and is widely used in studies involving Chinese populations [48,49,51]. The MoCA Full Chinese (Beijing) Version 7.1 was the official Beijing version published on the MoCA website [52]. MoCA scores were adjusted according to participants’ education level, with one additional point added for individuals with 12 years of formal education or fewer. Nonverbal memory was further assessed using the delayed recall condition of the Rey-Osterrieth Complex Figure Test, while visuospatial functioning was evaluated through the Copy condition of the Rey-Osterrieth Complex Figure (ROCF-C) test [53]. To examine executive function and working memory, Part A of the Trail Making Test (TMTA) was administered [54]. Additionally, spatial rotation ability was assessed using a standardized Mental Rotation Test (MRT) [55]. These assessments provided a comprehensive cognitive profile for each participant prior to engagement in the virtual navigation experiment.

**Figure 1.**
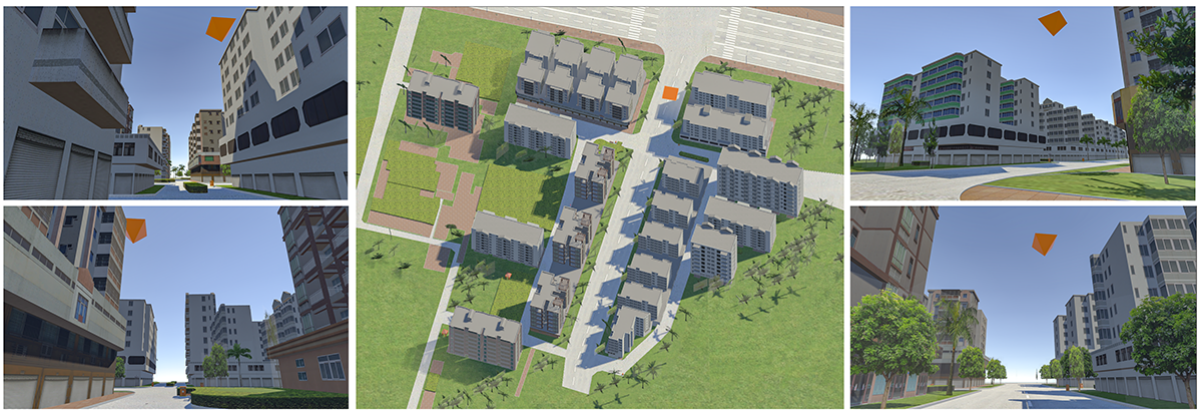
Pretraining phase: top-down view of the scene (middle) and views of the target from four different locations (left and right).

**Figure 2.**
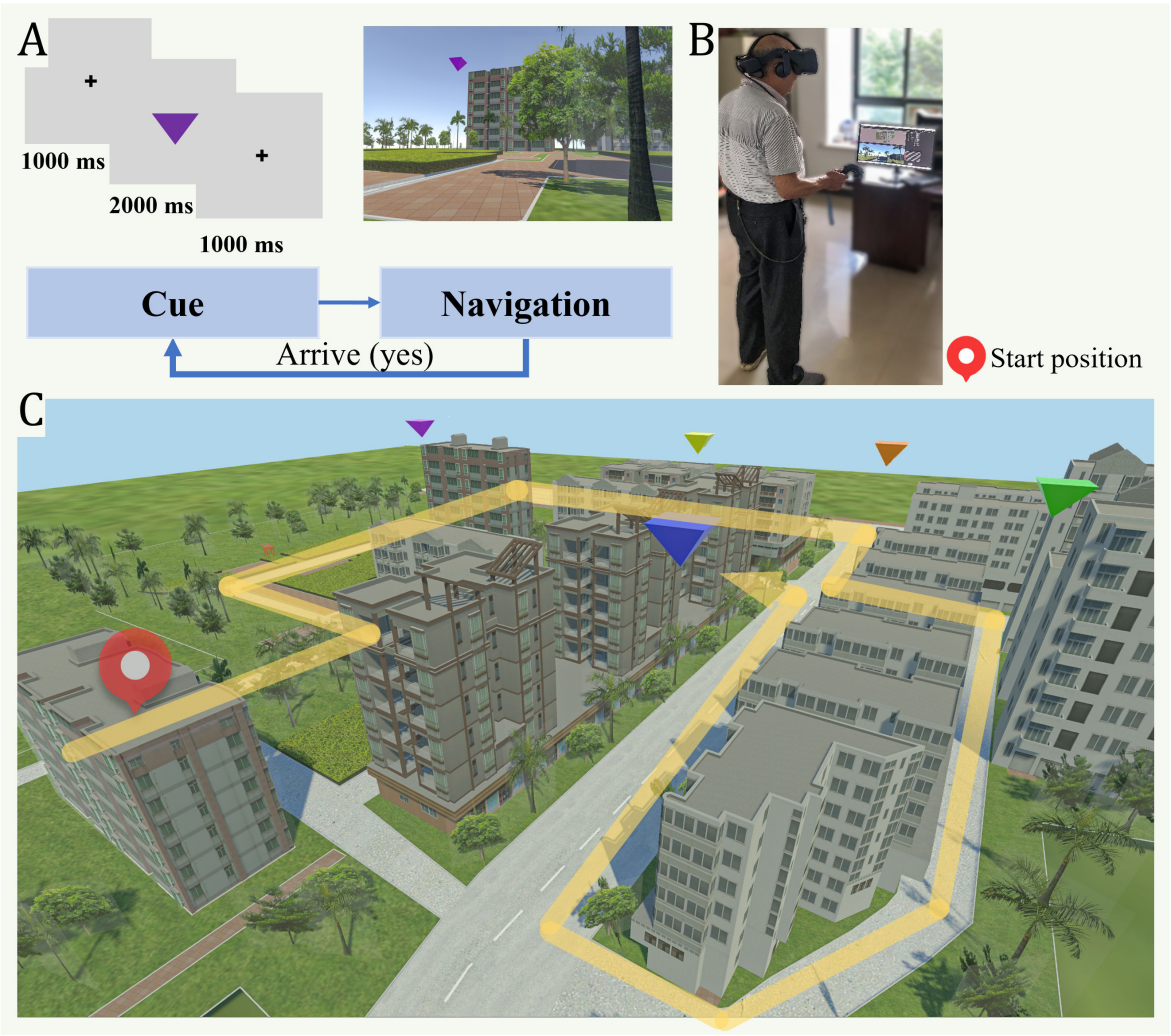
Formal experiment: (A) The sequence of a single trial. Following the presentation of a cue, participants were required to navigate to the target location. (B) Photograph of an older participant during the experiment. (C) Layout of the virtual city environment, including the shortest route planned to all target locations.

During the pre-experimental phase, identically shaped but differently colored target objects were placed at various locations within the VE (Figure 1). Participants were instructed to memorize the spatial locations corresponding to each colored object. The specific verbal instructions can be found in Multimedia Appendix 1. During the formal experimental phase, 5 target locations previously encountered during the training phase were used (Figure 2C). Target locations were presented in a fixed sequential order, allowing for consistent route comparisons across participants. Each trial began with a fixation cross (1000 ms), followed by the target cue (2000 ms), another fixation cross (1000 ms), and then participants were required to navigate to the designated target location (Figure 2A). Upon reaching the target, the current trial was concluded, and the next trial was automatically initiated. This sequence continued until all tasks were completed. Participants were instructed to navigate as quickly and accurately as possible (Figure 2B), and a congratulatory animation was displayed after each trial to prevent boredom.

### Environmental Structure Analysis

#### Space Syntax

Space Syntax provides a robust and systematic framework for examining the spatial arrangement of environments and the interrelationships between spaces. This methodology has been extensively applied to explore how spatial configurations affect human behavior [26,56,57], particularly how individuals encode, represent, and retrieve spatial information during navigation. In this study, both AMA and the VGA of the VE were carried out using the DepthmapX software (Space Syntax Laboratory).

#### Axial Map Analysis

**Figure 3.**
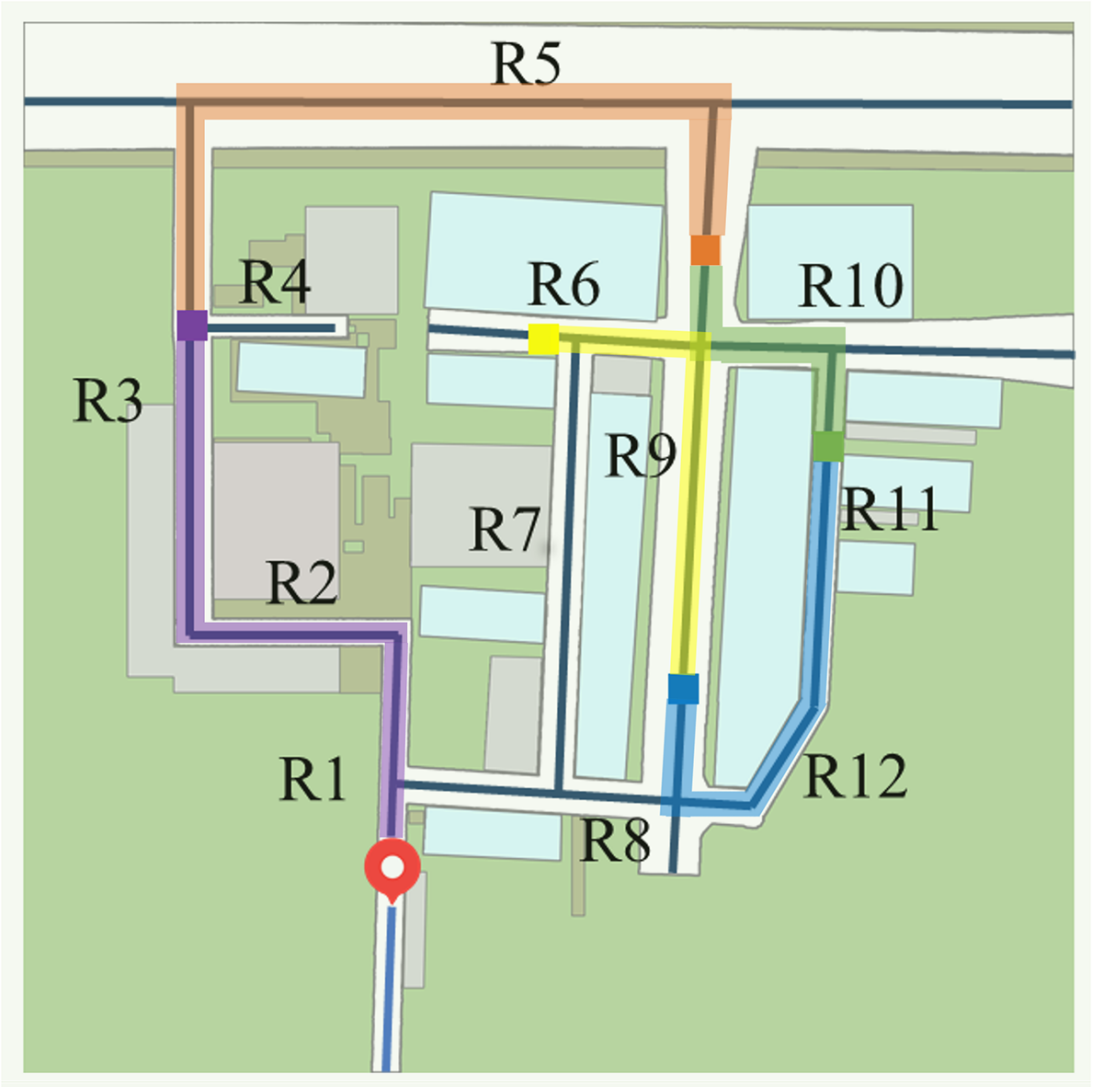
The axial map constructed from the 2D layout of the virtual environment street network.

The axial map provides a graphical representation of spatial structure, where streets within an urban environment are abstracted as axial lines, defined as the minimal set of longest straight lines of sight from a given location [23,58]. These lines collectively cover the entire space, capturing all possible movement paths. Based on the 2D layout of the VE street network, we constructed an axial map with a total of 12 axial lines delineated (Figure 3). For analytical purposes, key metrics such as axial integration, axial connectivity, and axial mean depth were calculated to characterize the accessibility and flow potential of individual environmental segments.

#### Visibility Graph Analysis

VGA provides an analytical approach for quantifying the spatial characteristics of an environment by assessing the extent to which any given point within the space is visible from any other point [59]. In this study, the structural layout of the VE was first modeled using AutoCAD. Subsequently, a 1 m×1  m grid was generated in DepthmapX to perform the analysis. This process enables the computation of key spatial metrics at each node, including visual integration, visual connectivity, and visual mean depth.

### Measurement

#### Metrics Setting

A set of behavioral and environment-based metrics was calculated in the virtual navigation game to evaluate the effectiveness of the game in assessing cognitive abilities and to examine age-related differences in navigation performance and sensitivity to environmental features. Spatiotemporal data were recorded for each participant at a fixed sampling frequency. Based on these data, the navigation behavioral metrics and environment-based navigation metrics were defined as follows.

#### Wayfinding, Transition, and Moving States

During navigation, we continuously recorded each participant’s spatial position $\left(x_{i},y_{i}\right)$ at timestamp $t_{i}$ . For each recorded point i (excluding the starting and ending points), the instantaneous speed was computed as the average of the velocity estimates derived from two adjacent time intervals.


$$v_{i}=\left(\frac{\sqrt{{\left(x_{i}-x_{i-1}\right)}^{2}+{\left(y_{i}-y_{i-1}\right)}^{2}}}{t_{i}-t_{i-1}}+\frac{\sqrt{{\left(x_{i+1}-x_{i}\right)}^{2}+{\left(y_{i+1}-y_{i}\right)}^{2}}}{t_{i+1}-t_{i}}\right)/2$$

The instantaneous speed series for each participant was then classified into 3 distinct categories using the k-means clustering algorithm, yielding low, medium, and high speed clusters. These clusters corresponded to meaningful navigation states: Wayfinding (low speed: searching, hesitation, or reorientation), Transition (medium speed: directional adjustment or preparatory acceleration), and Moving (high speed: continuous, goal-directed locomotion). This classification allowed us to segment the entire trajectory into functionally interpretable behavioral states.

#### Navigation Efficiency

Navigation efficiency is defined as the ratio of effective navigation time (moving time and transition time) to the total duration of the navigation game (navigation time).


$$Navigation\;efficiency=\frac{\left(Moving\;time+Transition\;time\right)}{Navigation\;time}$$

This metric serves as an indicator of the capability to navigate effectively within the environment. Higher values reflect a more streamlined and goal-oriented navigation process, characterized by reduced time spent on behaviors such as hesitation, reorientation, or uncertainty. By quantifying navigation efficiency, we can evaluate the extent to which individuals construct accurate cognitive maps and adopt effective spatial strategies during navigation tasks.

#### Experienced Axial Metrics

Axial analysis is derived from a representation of the environment using the fewest and longest lines of sight, which capture key structural characteristics of an urban street network. In this study, we used the axial values computed from the axial map shown in Figure 3. Three axial measures were considered: axial integration, which quantifies the accessibility of an axial line from the rest of the system; axial connectivity, which denotes the number of axial lines directly linked to a given line; and axial mean depth, which represents the arithmetic mean of the topological depths from each axial line to all others. To examine how participants’ navigation behavior was shaped by the underlying street configuration, we computed the average value of each metric along the participant’s trajectory, resulting in 3 experienced axial metrics: experienced axial integration (EAI), experienced axial connectivity (EAC), and experienced mean axial depth (EMAD).


$$EAI=\frac{1}{n}\sum_{i=1}^{n}I_{A}\left(x_{i},y_{i}\right)$$

$$EAC=\frac{1}{n}\sum_{i=1}^{n}C_{A}\left(x_{i},y_{i}\right)$$

$$EMAD=\frac{1}{n}\sum_{i=1}^{n}D_{A}\left(x_{i},y_{i}\right)$$

The point $\left(x_{i},y_{i}\right)$ denotes the coordinates of the participant at sample *i*, and $I_{A}$, $C_{A}$, and $D_{A}$ represent the integration, connectivity, and mean depth values of the axial line associated with that position. The variable n indicates the total number of sampled points along the participant’s trajectory.

#### Experienced Visual Metrics

**Figure 4.**
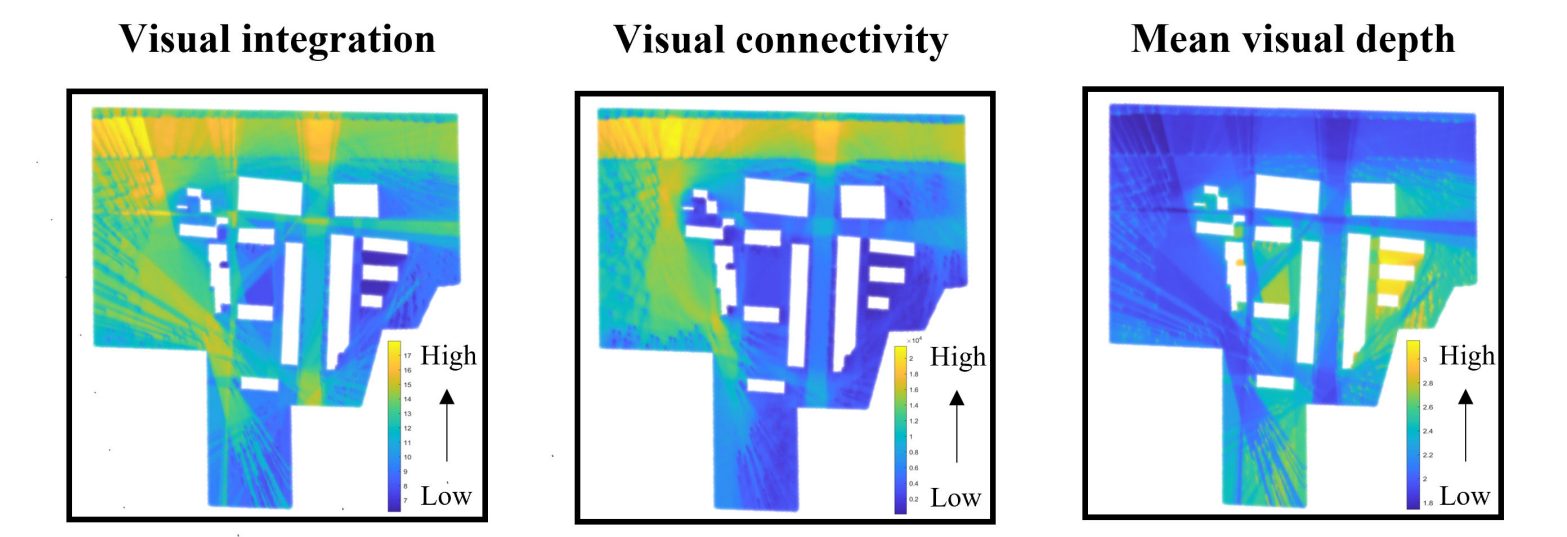
The visualization of the virtual urban spatial structure using visibility graph analysis.

VGA represents an environment as a grid-based visibility network, enabling the quantification of visual relationships between locations. The visual integration, visual connectivity, and visual mean depth values used in this study were derived from the VGA map shown in Figure 4. To examine how participants’ navigation behavior was influenced by visually accessible spatial properties, we computed the average value of each VGA metric along the participant’s trajectory, yielding experienced visual integration (EVI), experienced visual connectivity, and experienced visual mean depth.


$$EVI=\frac{1}{n}\sum_{i=1}^{n}I_{v}\left(x_{i},y_{i}\right)$$

$$EVC=\frac{1}{n}\sum_{i=1}^{n}C_{v}\left(x_{i},y_{i}\right)$$

$$EVMD=\frac{1}{n}\sum_{i=1}^{n}D_{v}\left(x_{i},y_{i}\right)$$

$I_{v}$, $C_{v}$, and $D_{v}$ represent visual integration, visual connectivity, and visual mean depth values associated with participants’ position $\left(x_{i},y_{i}\right)$ in the VGA grid.

### Statistical Analysis

Data from the experiment were processed using custom scripts developed in MATLAB, and all statistical analyses were performed using SPSS (IBM Corp). The normality of each variable was assessed through visual inspection of histograms and the Shapiro–Wilk test. Spearman’s rank correlation analyses were conducted to examine the associations between navigation-related behavioral measures and cognitive test scores. To evaluate age-related differences, independent-samples *t* tests were applied to variables that met normality assumptions, whereas the Mann-Whitney *U* test was used for variables that were not normally distributed. In addition, correlations between navigation efficiency and the various experienced spatial metrics were examined using Pearson correlations for normally distributed variables and Spearman rank correlations for nonnormally distributed variables.

## Results

### Participants

All 19 older participants and 18 young participants successfully completed the experiment without experiencing severe motion sickness. Table 1 summarizes the cognitive characteristics of the older group, including the means and SDs for various cognitive assessment scales. Notably, none of the participants had prior experience with VR technology, which reduced potential biases associated with equipment familiarity.

**Figure 5.**
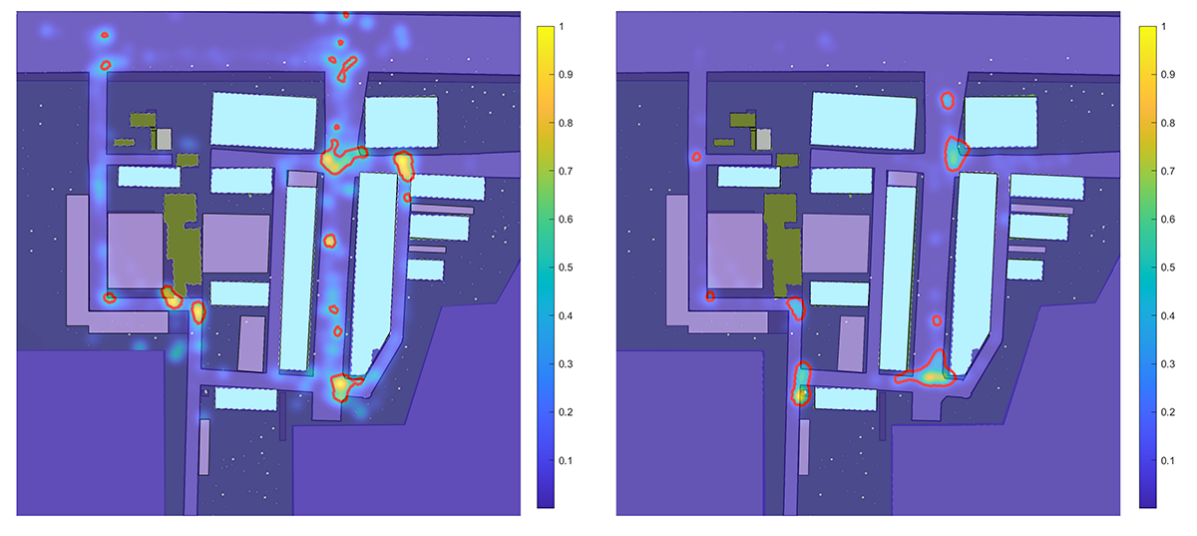
Spatial density distribution of wayfinding points (left: older adults; right: young adults).

After clustering the data, points labeled as wayfinding state exhibited clear spatial concentration and age-related differences (Figure 5). This spatial density map visualizes wayfinding patterns, offering an intuitive overview of participants’ spatial behavior based on spatiotemporal features. The red-outlined areas indicate the top 1% of regions with the highest density values, highlighting zones of intensified wayfinding activity within the VE.

**Table 1.** Characteristics of participants of different groups.

	Older adults	Young adults
Age (years), mean (SD)	71.79 (4.57)	26.50 (2.99)
Sex (male/female), n	14/5	10/8
Education >12 years (yes/no), n	11/8	18/0
VR^a^ experience (yes/no), n	0/19	0/18
MoCA^b^, mean (SD)	21.84 (5.47)	—^c^
MMSE^d^, mean (SD)	27.16 (3.60)	—
TMTA^e^, mean (SD)	62.06 (21.27)	—
ROCF-D^f^/ROCF-C^g^, mean (SD)	31.63 (4.59)/18.60 (5.62)	—
MRT^h^, mean (SD)	68.58 (13.62)	—

a VR: virtual reality.

b MoCA: Montreal Cognitive Assessment.

c Not available.

d MMSE: Mini-Mental State Examination.

e TMTA: Part A of the Trail Making Test.

f ROCF-D: delayed recall condition of the Rey-Osterrieth Complex Figure.

g ROCF-C: Copy condition of the Rey-Osterrieth Complex Figure.

h MRT: Mental Rotation Test.

### The Correlation Between Navigation Behavior and Cognitive Abilities in Older Participants

Spearman rank correlation analysis was conducted to examine the relationships between navigation behavior metrics and cognitive assessment scores (Table 2). No significant correlations were observed between either distance or error and any of the cognitive scales. However, temporal-based navigation parameters performed well. Navigation time was significantly associated with both TMTA (*r*=0.503, *P*=.047) and the MRT (*r*=−0.526, *P*=.03). Wayfinding time showed strong correlations with several cognitive measures, including TMTA (*r*=0.716, *P*=.002), MoCA (*r*=−0.549, *P*=.02), ROCF-C (*r*=−0.588, *P*=.01), MMSE (*r*=−0.458, *P*=.046) and MRT (*r*=−0.751, *P*<.001). Additionally, navigation efficiency demonstrated significant associations with MoCA (*r*=0.495, *P*=.04) and ROCF-C (*r*=0.658, *P*=.003) scores, and particularly strong correlations with TMTA (*r*=−0.761, *P*=.001) and MRT (*r*=0.848, *P*<.001). These findings confirmed that cognitive abilities play a crucial role in influencing navigation efficiency, including navigation time and wayfinding time.

**Table 2.** The correlation between spatial navigation and cognitive scales.

	MoCA^a^	MMSE^b^	ROCF^c^-C	ROCF-D^d^	TMTA^e^	MRT^f^	Distance	Error	Navigation time	Wayfinding time
MMSE										
*r*	0.885^g^	1								
*P* value	<.001	—^h^								
ROCF-C										
*r*	0.470^i^	0.364	1							
*P* value	.049	.14	—							
ROCF-D										
*r*	0.344	0.084	0.603^j^	1						
*P* value	.16	.74	.008	—						
TMTA										
*r*	−0.663^j^	−0.496	−0.380	−0.081	1					
*P* value	.005	.05	.15	.76	—					
MRT										
*r*	0.409	0.301	0.701^j^	0.349	−0.591^i^	1				
*P* value	.09	.22	.001	.16	.02	—				
Distance										
*r*	−0.287	−0.201	−0.013	−0.122	−0.105	0.103	1			
*P* value	.25	.42	.96	.63	.70	.68	—			
Error										
*r*	−0.278	−0.098	0.049	−0.203	−0.07	0.313	0.873^g^	1		
*P* value	.26	.70	.85	.42	.80	.21	<.001	—		
Navigation time										
*r*	−0.399	−0.286	−0.356	−0.247	0.503^i^	−0.526^i^	0.655^j^	0.473^i^	1	
*P* value	.10	.25	.15	.32	.047	.02	.003	.047	—	
Wayfinding time										
*r*	−0.549^i^	−0.458^i^	−0.588^i^	−0.296	0.716^j^	−0.751^g^	0.426	0.216	0.920^g^	1
*P* value	.02	.06	.01	.23	.002	<.001	.08	.39	<.001	—
Navigation efficiency										
*r*	0.495^i^	0.445	0.658^j^	0.206	−0.761^j^	0.848^g^	−0.067	0.129	−0.709^j^	−0.913^g^
*P* value	.04	.06	.003	.41	.001	<.001	.79	.61	.001	<.001

a MoCA: Montreal Cognitive Assessment.

b MMSE: Mini-Mental State Examination.

c ROCF-C: Copy condition of the Rey-Osterrieth Complex Figure.

d ROCF-D: delayed recall condition of the Rey-Osterrieth Complex Figure.

e TMTA: Part A of the Trail Making Test.

f MRT: Mental Rotation Test.

g *P*<.001

h Not applicable.

i *P*<.05

j *P*<.01.

### Analysis of Age-Related Decline in Wayfinding Ability

**Figure 6.**
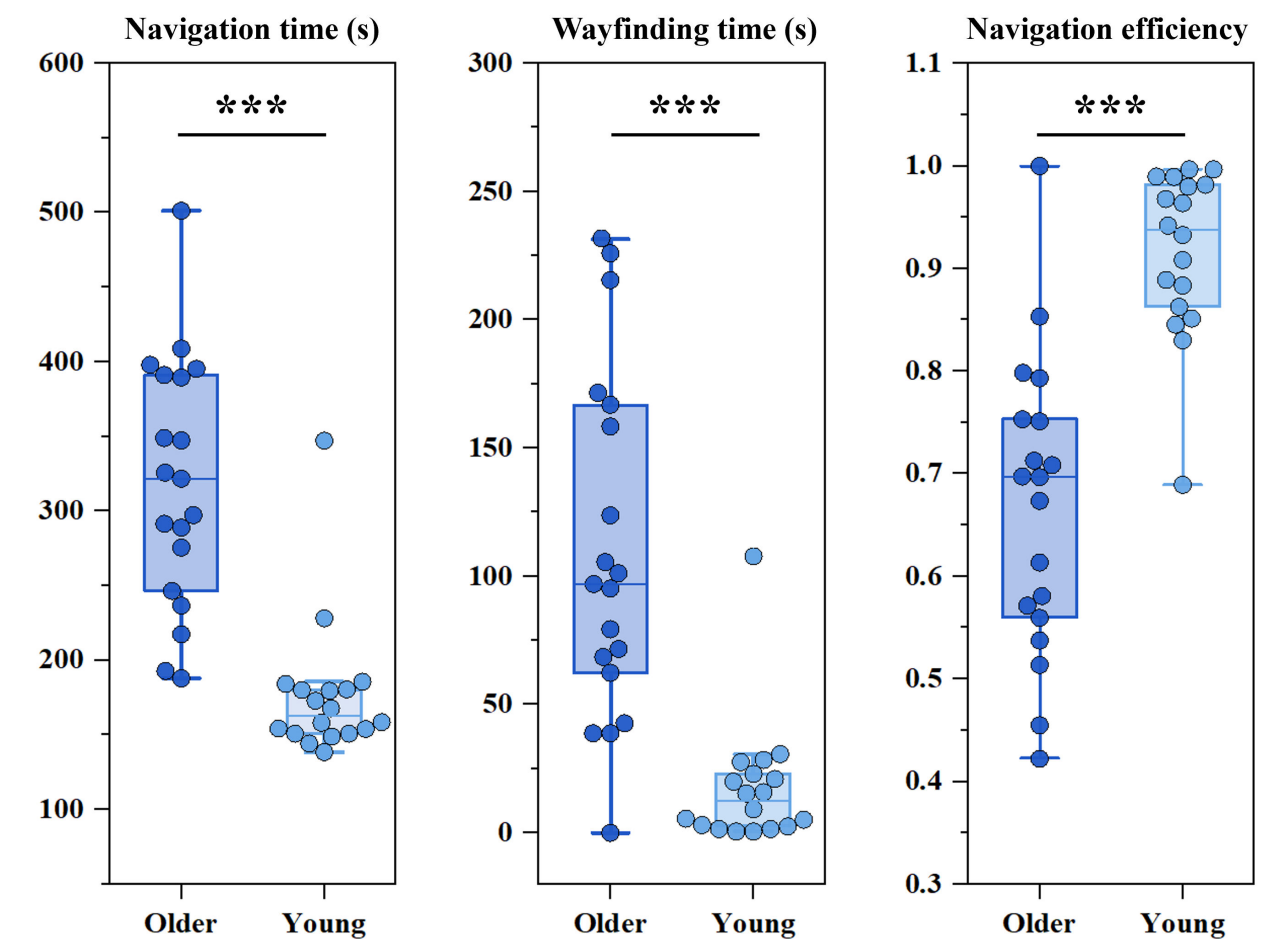
Box plots illustrating differences in navigation performance between older and younger participants.

The Mann-Whitney *U* test was used to assess differences in navigation performance between age groups (Figure 6). Compared to young participants, older adults, who exhibited significantly lower cognitive ability, demonstrated significantly longer navigation time (*z*=−4.771, *P*<.001) and wayfinding times (*z*=−4.315, *P*<.001), as well as reduced navigation efficiency (*z*=−4.285, *P*<.001).

**Figure 7.**
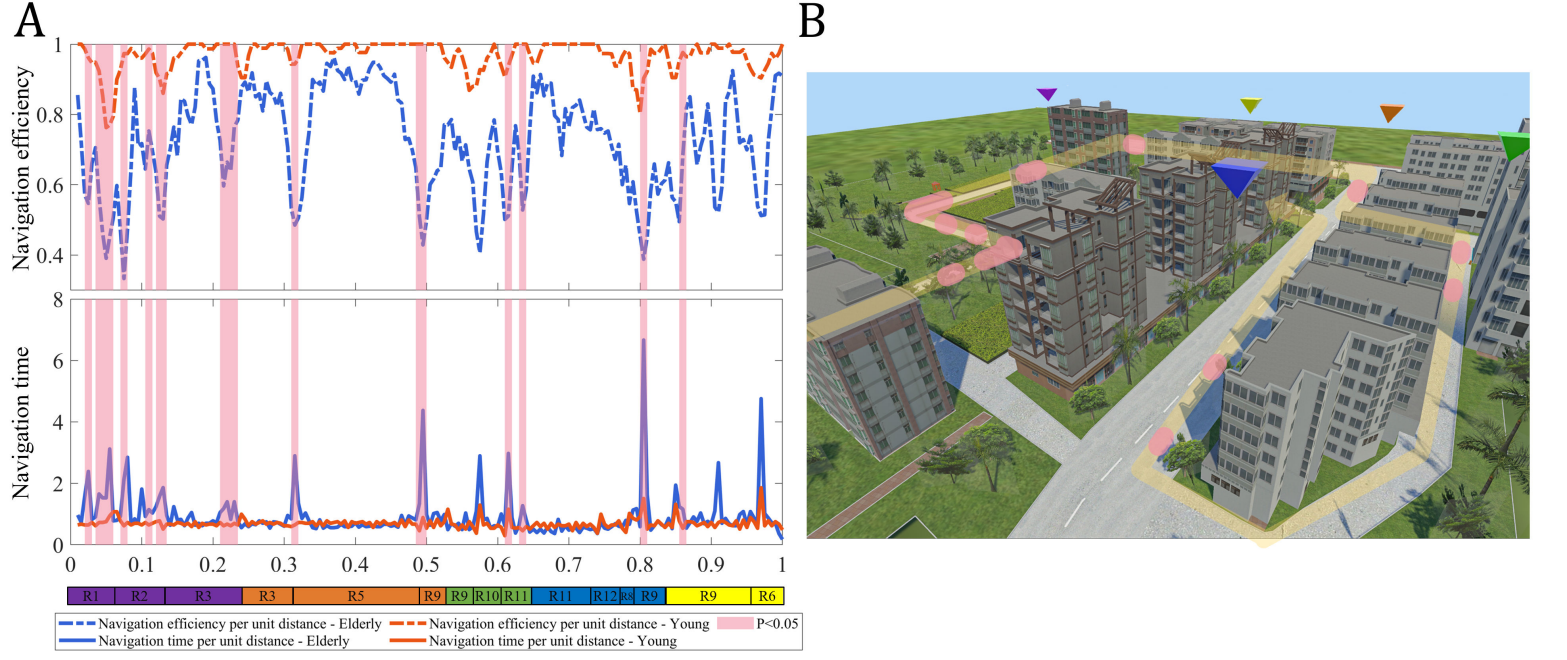
Comparison of the navigation process between older and younger participants during the formal experiment. (A) The x-axis represents the normalized value along the shortest distance across 5 consecutive trials, and the y-axis shows both navigation time and navigation efficiency. The colored bar at the bottom corresponds to the target color for each trial, and the labels R1, R2, ... indicate the axial lines that constitute the shortest navigation route for that trial. (B) Pink-shaded areas indicate route segments where significant differences in navigation efficiency were found between older and younger participants, as determined by permutation testing (*P*<.05).

The above metrics reflect participants’ overall wayfinding performance. However, navigation behavior is not solely influenced by internal factors such as cognitive ability. It also varies depending on the location of participants within the VE. Figure 7A illustrates differences in the navigation process across age groups during the formal experiment. To investigate this, participants’ spatial positions are mapped onto the shortest navigation routes (excluding trials involving route errors), and the time spent per unit distance is calculated for each individual. Based on these data, we plot curves of navigation time and navigation efficiency for each age group. Furthermore, a permutation test is conducted on navigation efficiency within each unit distance between older and young adults. The shaded regions on Figure 7B correspond to areas where significant differences are observed (*P*<.05) in Figure 7A, revealing spatial zones where age-related differences in navigation behavior are most pronounced. These results highlight age-related differences in spatial behavior and reinforce the utility of these behavioral metrics as sensitive indicators of cognitive function.

### Effects of Environmental Structure on Navigation Behavior in Different Age Groups

The influence of environmental characteristics on navigation performance is analyzed from the perspective of spatial configuration using both AMA and VGA. Between-group comparisons revealed significant age-related differences in several experienced spatial measures. Older adults showed higher EAI (*z*=–2.43, *P*=.01) and EVI (*t*=2.48, *P*=.02), whereas young adults exhibited higher EMAD (*z*=–2.13, *P*=.03) and EMVD (*t*=–2.49, *P*=.02). No significant group differences were found for experienced axial connectivity and experienced visual connectivity.

Correlation analyses further revealed distinct age-specific associations between navigation efficiency and experienced metrics. Among older adults, navigation efficiency showed a significant negative correlation with EVI (*r*=–0.48, *P*=.04) and a positive correlation with EMVD (*r*=0.49, *P*=.03). In contrast, young adults demonstrated strong associations with axial properties, with navigation efficiency negatively correlated with EAI (*r*=–0.64, *P*=.005) and positively correlated with EMAD (*r*=.72, *P*<0.001). Young adults also exhibited a significant positive relationship between navigation efficiency and EMVD (*r*=0.57, *P*=.01).

## Discussion

### Summary and Explanation of Findings

This study investigated how cognitive abilities and environmental structure jointly shape navigational performance in a virtual navigation game across different age groups. Consistent with prior research, our findings demonstrate that performance in navigation games, particularly navigation efficiency, is strongly influenced by individual cognitive capacity, as well as the spatial configuration of the environment. These results underscore the relevance of navigation behavior as an indicator of cognitive status and highlight the potential of VR-based navigation tasks as effective tools for cognitive assessment. Moreover, the insights gained from this work also provide valuable guidance for future urban design and navigation-game development.

The behavioral metrics used in this study, including traversal distance, number of errors, and navigation time, are widely used in human navigation research [34,60]. Spatiotemporal representation has long been essential for analyzing navigation behavior, and the present study extends conventional approaches by classifying participants’ navigation states using an individualized method. Navigation tasks are generally considered to consist of 2 main components [61]: locomotion and wayfinding. Locomotion refers to the physical execution of movement in the space, whereas wayfinding is involved in determining and planning a route to a desired destination [62]. Traditional approaches typically distinguish these components using fixed absolute speed thresholds [47,63]. To account for individual differences in navigation ability, we applied k-means clustering to each participant’s instantaneous speed distribution to derive personalized relative speed thresholds. k-Means is a classical clustering algorithm that iteratively assigns data points to the nearest cluster center [64]. To assess the reliability of this classification, clustering solutions with different numbers of clusters (*k*) were evaluated across all younger and older participants using the sum of squared errors (SSE) [65] and the silhouette coefficient [66] (Figure S3 in Multimedia Appendix 2). The SSE quantifies within-cluster variance by measuring the squared distance between each data point and its assigned cluster center, whereas the silhouette coefficient provides a complementary assessment of clustering quality by comparing consistency within clusters with separation between other clusters [67]. When *k*=3, the SSE curve showed a clear inflection point, indicating a markedly diminished rate of decrease in within-cluster variance. Meanwhile, the silhouette coefficient remained relatively high and exhibited more stable variance, suggesting an optimal balance between cluster cohesion and separation. In addition to these statistical indicators, the three clusters aligned well with meaningful navigation states observed in participants’ movement patterns (Figure S2 in Multimedia Appendix 3): (1) low-speed hesitation or searching, (2) medium-speed transitional adjustments, and (3) high-speed goal-directed movement. Taken together, both the quantitative metrics and the behavioral interpretability support the individualized clustering approach, which enabled the effective categorization of each sampled time point into low-, medium-, or high-speed states, thereby identifying regions within the environment where participants associated with wayfinding, transition, or continuous movement.

Effective spatial navigation relies on the integration of multiple abilities, including visual perception, spatial orientation, learning, and memory [68]. Wayfinding encompasses all of the ways in which people orient themselves in physical space and navigate from place to place. As anticipated, wayfinding ability, as a cognitive element of the navigation process [69], is effectively captured by parameters such as wayfinding time and navigation efficiency, both of which are shown to be sensitive indicators of cognitive capacity in the current study. As summarized in Table 2, cognitive scales including the MMSE, MoCA, ROCF-C, TMTA, and MRT exhibited robust correlations with these navigation metrics. Among these, the MoCA and MMSE are widely used for comprehensive cognitive assessment; the TMTA primarily reflects executive functioning; ROCF-C performance is sensitive to visuoconstructive and geometric processing abilities; and the MRT assesses mental rotation capacity. These cognitive functions collectively support navigation by contributing to processes such as initial route planning, continuous movement control, reorientation, and decision-making. SG-based VR navigation tasks elicit continuous, ecologically relevant behavior under dynamic task constraints, enabling the simultaneous assessment of multiple cognitive functions within an integrated behavioral context. Quantifying wayfinding performance provides an indirect yet meaningful reflection of an individual’s cognitive functioning [34]. Longer wayfinding times and lower navigation efficiency were associated with poorer cognitive performance in our older participants. Taken together, these findings highlight the potential of well-designed navigation games to serve as effective tools for cognitive assessment [70].

Human navigation in outdoor environments involves a series of distinctive behaviors, such as retracing steps, hesitation, and reorientation [71]. These behaviors become particularly evident in complex spatial settings where individuals must process environmental cues and make directional decisions. Wayfinding time serves as a key indicator for revealing navigation uncertainty, effectively pinpointing locations where external environmental factors impact individuals’ sense of directional uncertainty (Figure 4). We observe that prolonged wayfinding times were predominantly concentrated in the turning areas of the path and the starting or ending locations of each trial. These highlighted areas in the figure represent regions where participants are more likely to encounter decision-making bottlenecks, experiencing uncertainty in selecting the correct path [72]. Furthermore, integrating the analysis in Figure 6, we find significant differences in navigation performance between older and younger participants in certain regions.

Synthesizing findings from previous research, we propose that these results may be explained by age-related differences in navigation strategy preferences. Human navigation relies on 2 primary strategies: allocentric (survey-based) and egocentric (route-based). Younger adults tend to rely more on the allocentric strategy [18] and show greater flexibility in switching between allocentric and egocentric frames of reference [73]. However, aging is associated with a decline in allocentric navigation abilities, accompanied by a preferential shift toward egocentric strategies [18,21,22], which may contribute to less efficient route planning and increased reliance on familiar or visually accessible paths. These strategy differences align closely with the behavioral patterns observed in our study. Young participants adopted a more proactive and goal-directed approach: they typically scanned the environment at the beginning of each trial, extracted relevant spatial cues, and formed a planned route before moving. In contrast, older participants showed a stimulus-response navigation pattern [74,75], making decisions only upon arriving at intersections rather than planning ahead. This led to hesitation at nearly every decision point (Figure 6B), reflecting greater reliance on immediate cues and reduced anticipation of upcoming turns. Consequently, participants spent more time searching for landmarks or other environmental cues, leading to decreased navigation performance [76,77]. Such behaviors are consistent with age-related declines in spatial working memory and executive function [78], which limit their ability to efficiently integrate spatial cues.

To explore how environmental structure influences navigation across age groups, we used line-based (AMA) and grid-based (VGA) analytic approaches. Both methods quantify spatial configuration and offer complementary perspectives on how spatial structure shapes navigation behavior. Within this framework, integration serves as a core space syntax metric capturing the degree to which a path is connected to its surrounding environment [13,29]. In this study, experienced integration reflects the actual spatial integration values of the segments that participants traversed, thereby characterizing the dynamic interplay between environmental structure and navigational behavior. Older adults exhibited significantly higher EAI and EVI than young adults, suggesting a tendency to navigate toward more integrated areas rather than taking shorter or more direct routes. Prior studies have shown that spending more time in highly integrated areas facilitates the formation of accurate cognitive maps [13]. With aging, older adults require more time to form cognitive maps and encode spatial information [79], which may partly explain their tendency to remain within highly integrated axial regions during navigation. From another perspective, although integration is the normalization form of mean depth [25], the two measures capture different aspects of spatial structure. Mean depth quantifies the average topological steps from a location to all others, with higher values indicating deeper and harder-to-reach positions. Older adults showed lower EMAD and EMVD values, suggesting a tendency to remain within relatively shallower, more accessible areas.

**Figure 8.**
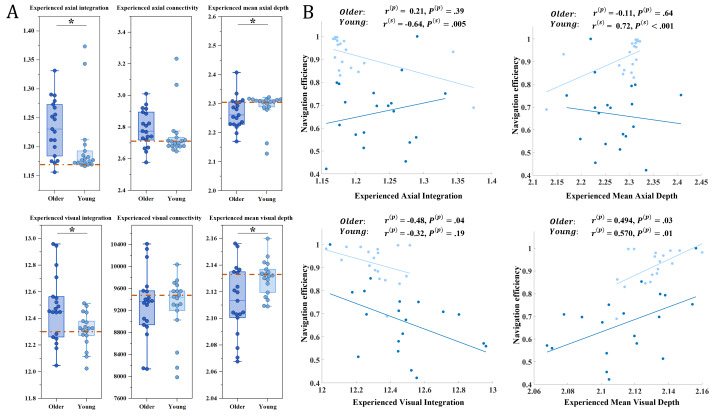
Experienced metrics across age groups and their associations with navigation efficiency. (A) Group differences in 6 experienced spatial metrics, experienced axial integration (EAI), experienced axial connectivity, experienced mean axial depth (EMAD), experienced visual integration (EVI), experienced visual connectivity, and experienced visual mean depth (EVMD) are shown for older adults (dark blue) and young adults (light blue). The brown dashed line represents the experienced metric obtained from the shortest path. (B) Correlations between navigation efficiency and 4 key experienced metrics (EAI, EMAD, EVI, and EVMD) are presented separately for older adults (dark blue) and young adults (light blue). Pearson correlations are denoted by (p) and Spearman correlations by (s).

In our navigation game, all participants were instructed to reach the target location as quickly as possible. By comparing each group’s experienced metrics with those of the shortest path (Figure 8A), we found that the experienced metrics of younger participants closely matched the optimal path. This pattern is consistent with behavioral observations showing that young participants, driven by the goal of rapid completion, tended to select more direct and efficient routes. Correlation analyses further support these environment-mediated navigation patterns. Among young adults, EAI was negatively correlated with navigation efficiency, indicating that more movement through globally integrated axial lines was associated with increased wayfinding behaviors (eg, hesitation, pausing, reorientation), thereby reducing overall efficiency. For older adults, EVI showed a significant negative correlation with navigation efficiency, suggesting that more extensive traversal within visually integrated regions corresponded to heightened hesitation or searching behaviors. The results for EMAD and EMVD exhibited similar trends: achieving higher navigation efficiency and shorter routes requires participants to strategically decide whether to traverse spatially advantageous or disadvantageous areas. Younger adults tended to accept navigating through deeper or less accessible regions to optimize route efficiency [56], whereas older adults were more likely to remain within visually accessible areas, even when these were not part of the shortest path.

This subjective tendency in navigational behavior indicates that participants do not passively respond only to environmental structure; rather, their navigational choices also reflect intentional, strategy-driven preferences [13]. Such findings point to a bidirectional relationship between spatial configuration and navigation behavior, where environmental structure shapes movement patterns, and individuals’ strategic preferences, in turn, influence the parts of the environment they traverse. Understanding whether environmental properties or individual behavior play the dominant role in guiding navigation will require further empirical investigation.

Additionally, our findings extend this literature by demonstrating age-specific patterns in the relationship between experienced metrics and navigation efficiency. Navigation efficiency in younger adults was strongly associated with line-based (AMA) metrics such as EAI and EMAD, whereas older adults showed stronger associations with grid-based (VGA) metrics, such as EVI and EMVD. These differences can be meaningfully interpreted through the lens of allocentric and egocentric navigation strategies. Younger adults tend to rely more on allocentric representations, enabling them to rapidly extract structural information from the environment, integrate axial connectivity, and flexibly switch between global structure and local visual information. This cognitive flexibility allows them to traverse deeper or less accessible areas. In contrast, aging is associated with an increased reliance on egocentric navigation. Older adults become more dependent on the visual field and immediate cues, which aligns with their stronger correlation between navigation efficiency and visual-based experienced metrics (EVI and EMVD). Their tendency to remain within visually accessible, highly integrated regions suggests that they prioritize environments that offer clear visual guidance and reduced cognitive demands [14].

Together, space syntax highlights the fundamental role of spatial configuration in shaping navigation behavior, and our findings further show that its influence varies with age, ultimately affecting navigation performance. These insights have important implications not only for developing more effective cognitive assessment tools but also for designing age-friendly urban environments that support safe, efficient, and accessible navigation. Strengthening the coordination and integration of street space is essential for urban street-design guidelines and road-network optimization [80]. Through axial and grid-based analyses, space syntax provides a powerful framework for identifying high integration. Such information can guide the placement of landmarks, directional signage, and other environmental cues that facilitate cognitive map formation and enhance navigability for diverse user groups. Moreover, because spatial integration is closely tied to navigation difficulty [29], purposeful modulation of integration levels can help calibrate the cognitive demands of navigation tasks. From an SG perspective, the majority of participants verbally reported a high level of immersion and task engagement, suggesting that the SG-based framework effectively supported sustained interaction with the spatial environment. By integrating principles of spatial configuration into an immersive VR navigation game, this approach may provide promising opportunities for the development of gamified tools for cognitive assessment, training, and rehabilitation, particularly for individuals with spatial or cognitive impairments.

### Limitations and Future Work

This research highlights the critical role of cognitive abilities and spatial configuration in shaping navigation behavior. However, several limitations should be acknowledged. First, the participant sample may not fully represent the broader population. The young participants were primarily between 20 and 30 years of age, whereas the older group ranged from 65 to 85 years, leaving a substantial gap between ages 30 and 65 years, which was not included. This omission limits the generalizability of the findings, as individuals in midlife may exhibit distinct navigation patterns and cognitive characteristics that were not captured in the current analysis [72]. Second, the individualized k-means clustering approach adopted in this study is particularly suited to navigation games characterized by relatively stable movement speeds. It may be less appropriate for tasks involving pronounced, irregular, or continuous speed transitions, in which clear and interpretable cluster boundaries are difficult to establish. Moreover, although a 3-cluster solution showed good behavioral interpretability within the current navigation game, the generalizability of this clustering scheme to other navigation games or more complex real-world environments remains to be validated in future studies. Third, discrepancies between virtual and real-world navigation are unavoidable [76]. The absence of environmental features such as directional signs, combined with the lack of natural physical movement, may influence how spatial information is encoded, updated, and retrieved [81]. Finally, the study did not explicitly account for the influence of demographic factors such as gender, educational background, or geographic and cultural experience. Ignoring these variables could obscure important individual differences and interactions relevant to wayfinding performance.

Future research should aim to address these limitations by recruiting a more demographically diverse participant sample, including middle-aged individuals, and by designing VEs with greater ecological validity. Incorporating navigation methods that more closely approximate natural physical movement may yield richer behavioral data and uncover additional nuances in navigational performance. Additionally, systematically examining the role of experienced metrics could provide deeper insights into urban design, spatial planning, and navigation-aid development in both virtual and real-world contexts. From a rehabilitation perspective, the game demonstrates promising potential for virtual navigation tasks as a controlled and adaptable platform for personalized cognitive assessment and training. Further research could investigate the incorporation of adaptive task difficulty and longitudinal monitoring into VR-based rehabilitation, thereby supporting the maintenance or recovery of spatial and cognitive functions.

### Conclusions

This study provides quantitative evidence for how environmental configuration and cognitive abilities jointly shape navigational behavior in VEs. By applying an individualized k-means clustering approach, we extracted key behavioral indicators, such as wayfinding time and navigation efficiency, that proved sensitive to cognitive decline. This method offers a more personalized and potentially more effective framework for assessing cognitive function through navigation tasks. Through spatial analysis, we further identified age-specific regions of wayfinding difficulty and quantified the extent to which environmental structure influences navigation behavior. These findings reveal distinct navigation patterns between younger and older adults and underscore the value of integrating behavioral analytics with spatial-syntax metrics. Overall, the results lay an important foundation for future applications in clinical cognitive assessment and rehabilitation, as well as in the development of age-friendly urban environments.

## Supplementary material

10.2196/83128 Multimedia Appendix 1 Instructions presented on the desktop during each phase of the navigation game.

10.2196/83128 Multimedia Appendix 2 (A) and (B) show the sum of squared errors and silhouette coefficient, respectively, for younger and older adults across different values of k.

10.2196/83128 Multimedia Appendix 3 (A) and (B) show all instantaneous speed samples from older and young participants, respectively. Blue, green, and yellow points represent low-, medium-, and high-speed clusters, respectively, and black points indicate the cluster centroids. (C) and (D) present the probability distributions of navigation speed for older and younger participants, respectively, with dark blue representing the group mean and light blue indicating individual distributions.

## Abbreviations

AMA: axial map analysis
EAI: experienced axial integration
EMAD: experienced mean axial depth
EVI: experienced visual integration
MMSE: Mini-Mental State Examination
MoCA: Montreal Cognitive Assessment
MRT: Mental Rotation Test
ROCF-C: Copy condition of the Rey-Osterrieth Complex Figure
SG: serious game
SSE: sum of squared errors
TMTA: Part A of the Trail Making Test
VE: virtual environment
VGA: visibility graph analysis
VR: virtual reality
