# Supplementary figures and images for: Analysis of Spatiotemporal Features in a Virtual Navigation Game Across Different Age Groups: Quantitative Research
Source: JMIR Serious Games. 2026 Apr 2;14:e83128. doi: 10.2196/83128 (PMC13046224; doi:10.2196/83128)

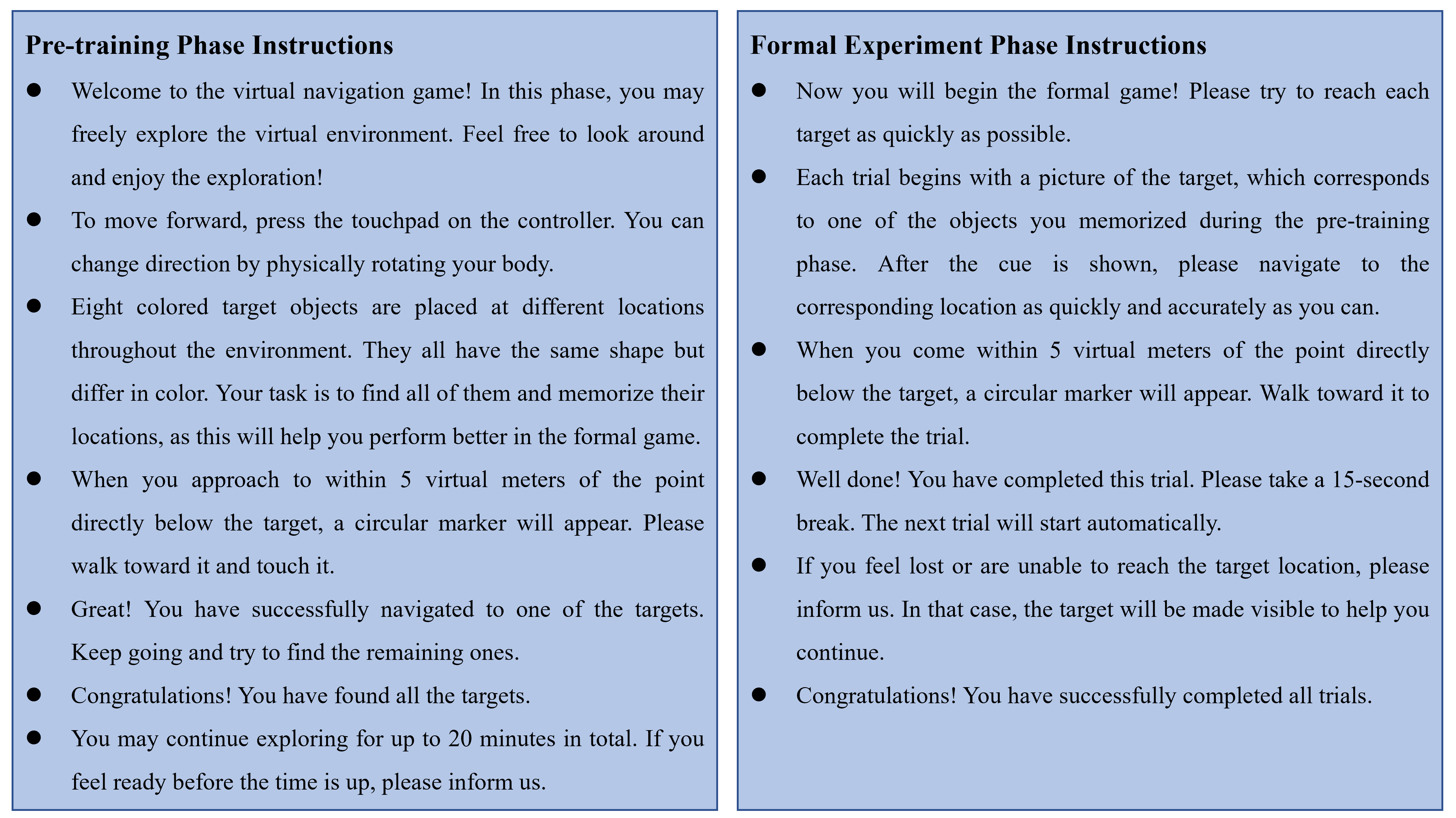

Supplement: Multimedia Appendix 1 [file games-v14-e83128-s001.png]

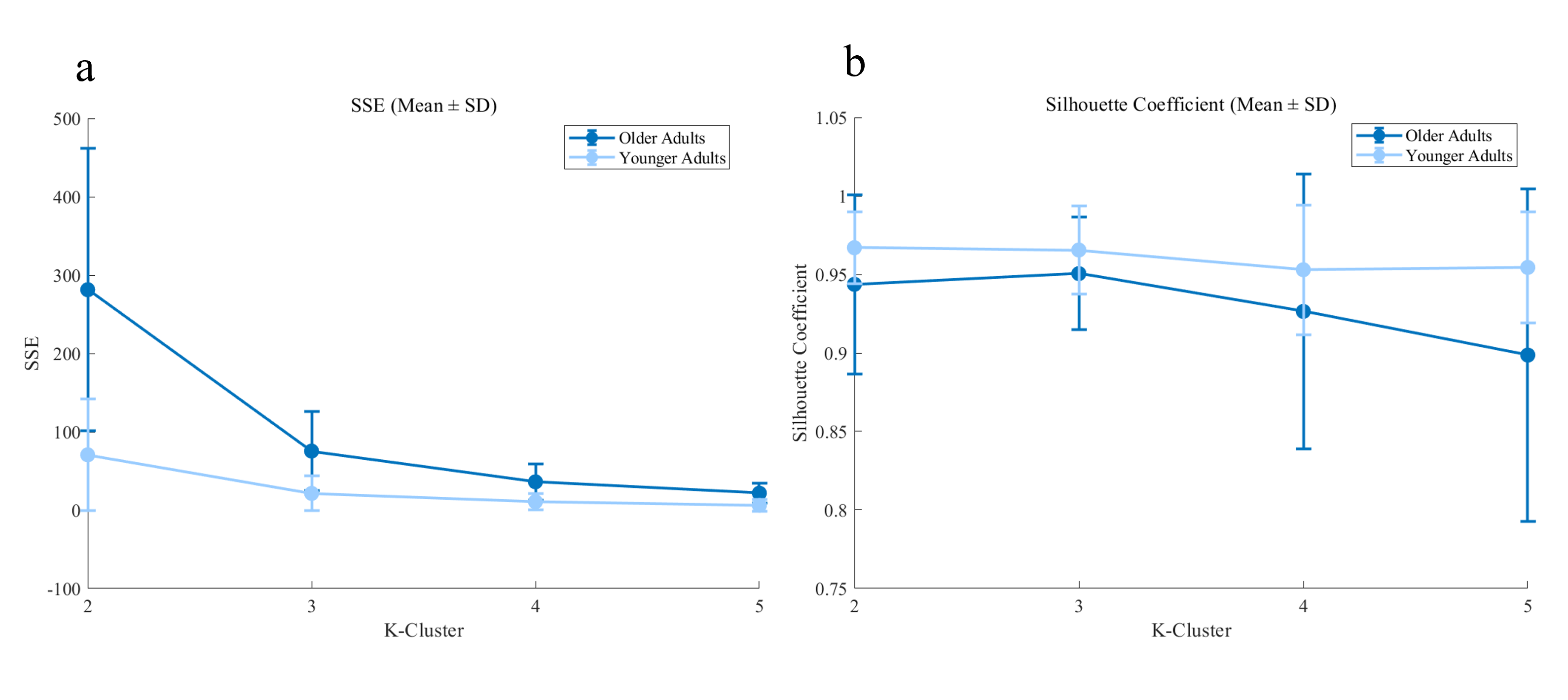

Supplement: Multimedia Appendix 2 [file games-v14-e83128-s002.png]

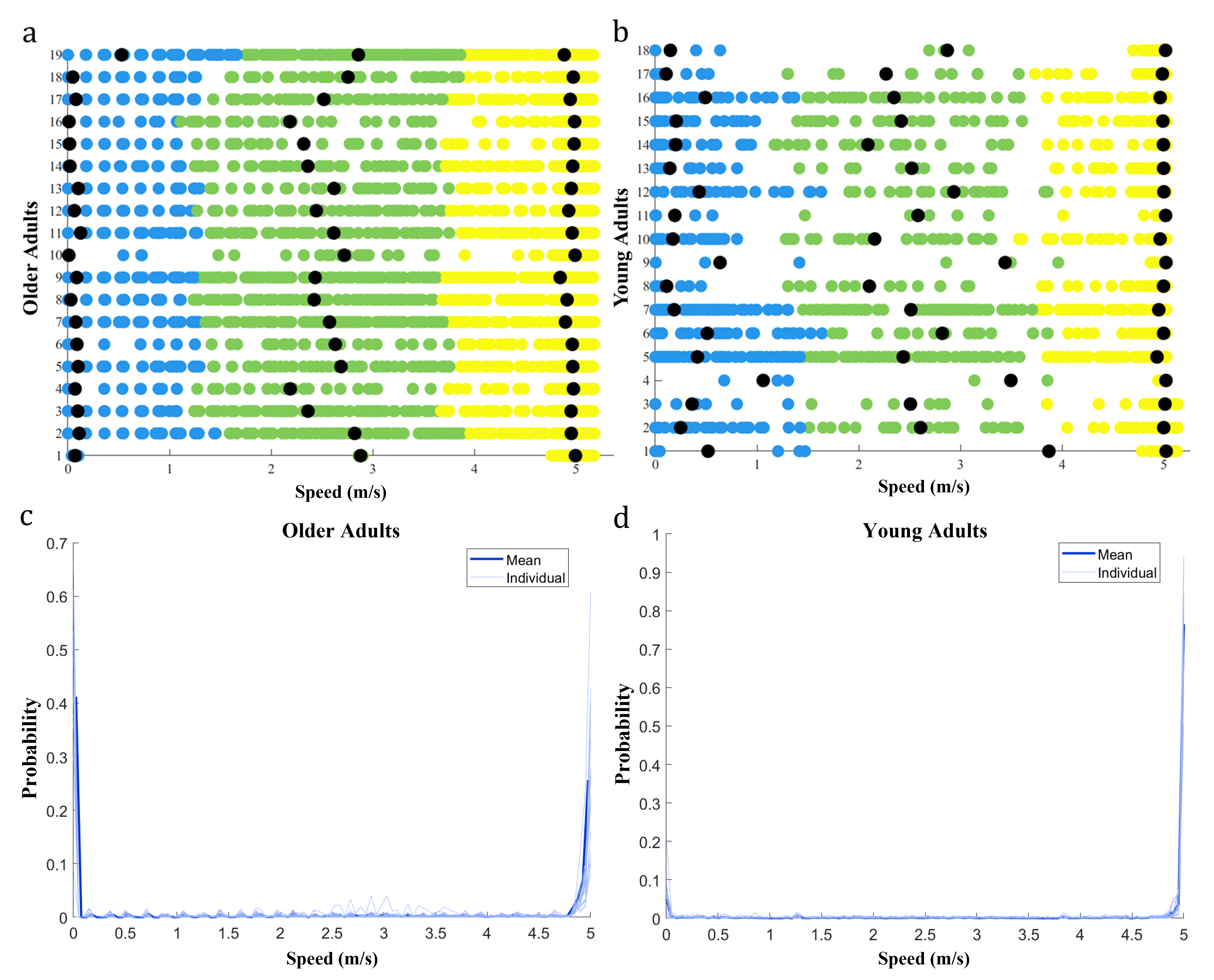

Supplement: Multimedia Appendix 3 [file games-v14-e83128-s003.png]
